# DNA methylation-driven genes in hepatocellular carcinoma patients: insights into immune infiltration and prognostic implications

**DOI:** 10.3389/fmed.2025.1520380

**Published:** 2025-04-28

**Authors:** Zhi Zhang, Tongling Zhao, Weida Meng, Jiahao Chen, Chengyi He, Xing Sun, Hai Huang

**Affiliations:** ^1^Department of Hepatobiliary Surger, Guangxi Medical University Affliated Wuming Hospital, Nanning, China; ^2^Collaborative Innovation Centre of Regenerative Medicine and Medical BioResource Development and Application Co-Constructed by the Province and Ministry, Guangxi Medical University, Nanning, China; ^3^Department of Hepatobiliary Surgery, The Fifth Affiliated Hospital of Guangxi Medical University, Nanning, China

**Keywords:** hepatocellular carcinoma, bioinformatics, TCGA, WGCNA, prognosis

## Abstract

**Background:**

Hepatocellular carcinoma (HCC) poses a significant global burden as a highly prevalent and life-threatening malignant tumor that endangers human life and wellbeing. The purpose of this study was to examine how DNA methylation-driven genes impact the prognosis of HCC patients.

**Methods:**

Differentially expressed genes from The Cancer Genome Atlas, GSE76427, GSE25097 and GSE14520 datasets were collected to perform differential expression analysis between HCC patients and controls. Weighted gene coexpression network analysis (WGCNA) was subsequently performed to create coexpression modules for the DEGs. Then, ssGSEA was employed to investigate the infiltration of immune cells in HCC. Enrichment analysis and methylation were carried out for the module genes. We utilized Kaplan–Meier survival analysis to assess patient prognosis.

**Results:**

Eight coexpression modules were identified via WGCNA for 1927 upregulated and 1,231 downregulated DEGs, after which the hub genes of the modules were identified. Module 5 had high immune infiltration, and the hub gene SCAMP3 was positively associated with Tcm. Module 3 exhibited a low level of immune infiltration, and the expression of the hub gene HCLS1 was negatively correlated with T cells and dendritic cells. Furthermore, we obtained five hub genes (BOP1, BUB1B, NOTCH3, SCAMP3, and SNRPD2) as methylation-driven genes. BOP1 and BUB1B were found to be correlated with unfavorable overall survival in patients with HCC.

**Conclusion:**

HCLS1 and SCAMP3 are associated with immunity, whereas BOP1 and BUB1B are modified by methylation and may serve as prognostic markers for HCC.

## Introduction

Hepatocellular carcinoma (HCC) ranks as the fifth most prevalent cancer globally and is the third leading cause of cancer-related fatalities (1, 2). HCC has a high mortality rate, and its incidence is increasing (3). The 5 years survival rate is only 18%, mainly because the majority of patients are diagnosed at an advanced stage (4). According to the SEER registration project, the incidence of HCC is projected to increase until 2030 (5). Currently, liver resection (LR), liver transplantation (LT), and percutaneous radiofrequency ablation (RFA) are considered effective methods for treating different stages of HCC (6). However, the high recurrence rate of HCC remains a key problem in LR (7). In addition to tumor size, the efficacy of RFA is also limited by tumor location (8, 9).

Despite numerous investigations, most patients suffering from HCC face a discouraging prognosis. The interaction between different immune cells and tumor cells has garnered significant attention in recent years. The HCC microenvironment, which has an immune-rich background, is a promising target for immunotherapy (10). The development of various novel immunotherapy techniques, including immune checkpoint blockade and chimeric antigen receptor T-cell therapy, has led to fresh optimism in treating HCC (11, 12). Hence, immune cell-associated genes could be considered possible targets for therapy.

Moreover, the atypical methylation of DNA in cancer can cause the suppression of tumor suppressor genes or the deregulation of genes, ultimately facilitating tumor promotion, a critical step in cancer development (13, 14). Accumulating evidence indicates that DNA methylation abnormalities can affect clinical outcomes in HCC patients (15, 16). DNA methylation has become a key factor associated with the diagnosis, treatment, prognosis, and malignant progression of HCC (17). Kisiel also found that TSPYL5 and SPINT2 were hypermethylated in HCC tissues (18). The expression of TSPYL5 was found to effectively separate HCC from corresponding non-tumor adjacent tissues in the same patients (19, 20). However, the link between DNA methylation-driven genes and the diagnosis and prognosis of HCC is not fully understood.

In this study, we investigated the significant methylation markers that are relevant to patient prognosis. We examined differentially expressed genes between tumor and non-tumor tissues from patients with HCC in publicly available datasets. Afterward, the researchers evaluated the characteristics of immunocyte infiltration, conducted enrichment analysis, and examined methylation regulation. The findings from this study could lead to the identification of valuable biomarkers and targets for potential therapies that could affect the occurrence and prognosis of patients with HCC.

## Materials and methods

### Data sources and differentially expressed genes

We obtained gene expression profiles from two distinct sources, Gene Expression Omnibus (GEO) datasets^1^ (21) and The Cancer Genome Atlas (TCGA)^2^ (22). The GSE76427 dataset included 115 HCC tissues and 52 adjacent non-tumor tissues (ANTTs), while the GSE25097 dataset included 268 HCC tissues and 243 ANTTs. In addition, GSE14520 comprised 222 HCC samples and 212 ANTTs. Each dataset was individually normalized using the robust multi-array average (RMA) method. The outlier detection was performed using the Grubbs’ test, and samples with extreme values were excluded from further analysis. The TCGA cohort included 369 liver cancer samples and 50 control samples. We utilized the DESeq2 (23) R package to standardize the data and perform differential expression analysis between the liver cancer and control samples in the TCGA. The differentially expressed genes (DEGs) between HCC and ANTT samples in the GEO dataset were screened using the limma package (24). A threshold of *p* < 0.05 was used to indicate a statistically significant difference. This threshold is commonly employed in bioinformatics and genomics studies to balance false positives and negatives, ensuring reliable detection of DEGs.

### Weighted gene coexpression network analysis (WGCNA)

The coexpression network was constructed using the R package WGCNA (25) with the intersected DEGs from four datasets. Briefly, the expression profiles were used to calculate Pearson’s correlation matrices. The ideal soft threshold was determined and used to create a weighted adjacency matrix. Then, a topological overlap measure (TOM) matrix was obtained, and modules were identified using hierarchical clustering with minModuleSize = 30 and mergeCutHeight = 0.25. Additionally, the hub gene of each module was determined based on the degree of connectivity of genes within the module (26). Specifically, we used network analysis methods to determine the importance of each gene in the module by calculating its correlation with other genes in the module. We have selected the gene with the highest correlation within the module as the hub gene of the module. We used the pROC R package (27) to plot receiver operating characteristic (ROC) curves, which were used to evaluate the clinical diagnostic ability of the hub genes.

### Single-sample gene set enrichment analysis (ssGSEA)

The marker gene set for congenital and adaptive immune cells, include 24 immune cell types was obtained (28), where ssGSEA was used to evaluate the infiltration of immune cells in HCC though the GSVA R package (29). ssGSEA method can calculate the enrichment score of 24 immune cell types in each sample, which reflects the activity level of that immune cell type in each sample. Tumors with varying infiltration patterns of immune cells were grouped using hierarchical clustering. The correlation between the ssGSEA score of immune cells and the expression of module genes was calculated using Pearson correlation analysis.

### Gene set enrichment analysis (GSEA)

The clusterProfiler package (30) was used to perform the enrichment analysis of the module genes. A *P* value less than 0.05 was used as the cutoff for both Gene Ontology (GO) functional analysis and Kyoto Encyclopedia of Genes and Genomes (KEGG) pathway analysis. The gene set enrichment analysis of the module genes was carried out through the use of the GSEA tool^3^.

### Methylation analysis

The methylation characteristics were obtained from the TCGA dataset using the Illumina Human Methylation 450K DNA Analysis BeadChip assay. The ChAMP package (31) was used to process the data and identify differentially methylated positions (DMPs).

Overall survival (OS) was estimated using Kaplan–Meier (K-M) survival analysis. The results with *P* < 0.05 were considered to indicate statistical significance.

### Correlation analysis between BOP1 or BUB1B and immune checkpoints

The associations between BOP1 or BUB1B and immune checkpoints were assessed through Pearson correlation analysis. The tumor purity (32) of the HCC cells was assessed, and the correlation between BOP1 or BUB1B expression and tumor purity was calculated.

### Statistical analysis

In the study, all the bioinformatics analyses were performed by bioinformatics cloud platform^4^.

## Results

### Genes that are differentially expressed in patients with HCC

**FIGURE 1:**
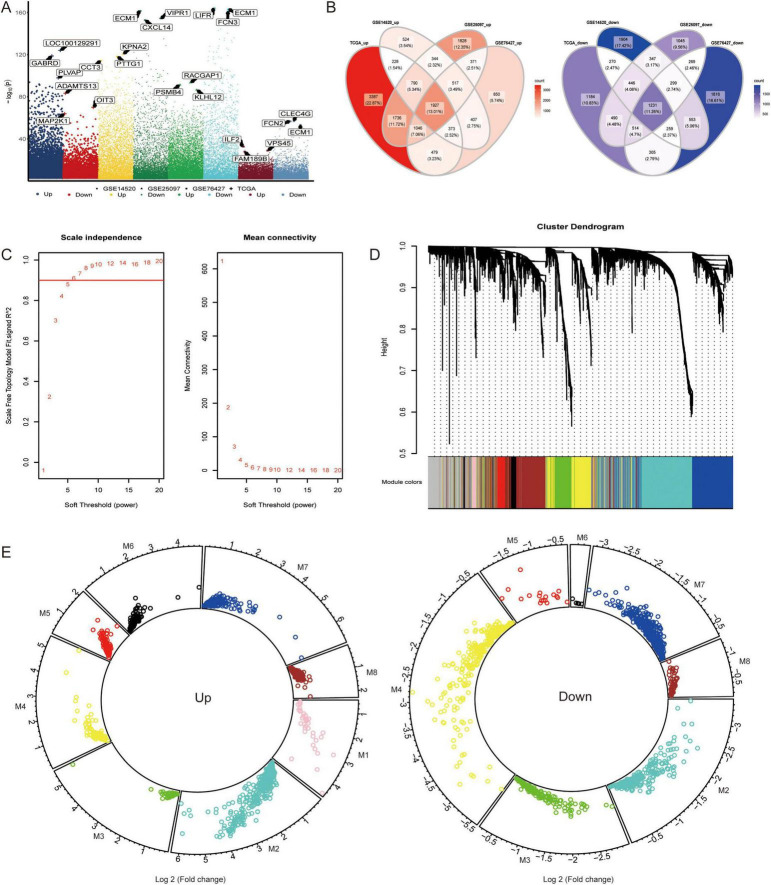
Coexpression of hepatocellular carcinoma-related genes. **(A)** Differentially expressed genes (DEGs) were identified between the hepatocellular carcinoma (HCC) and control groups in the TCGA, GSE14520, GSE25097, and GSE76427 datasets. The genes were considered significantly differentially expressed with *p* < 0.05. **(B)** The intersection of DEGs among the four datasets was determined using a Venn diagram to identify commonly upregulated and downregulated genes. **(C)** The soft threshold power for Weighted Gene Co-expression Network Analysis was determined based on the scale-free topology criterion. **(D)** The DEGs were clustered into eight modules based on hierarchical clustering of the gene expression data. Module significance was assessed using the K-means clustering algorithm, and the number of clusters was determined based on the silhouette score. **(E)** The upregulated or downregulated DEGs within each module were determined by calculating the average log2 fold change across datasets and tested for significance using *t*-test (*p* < 0.05).

To determine the genes related to HCC patients, DEGs were obtained between the four groups of HCC patients and controls (TCGA, GSE76427, GSE25097, and GSE14520) (Figure 1A). Among these genes, 1,927 upregulated genes and 1,231 downregulated genes were present at the intersection of the four groups of DEGs (Figure 1B). The progression of HCC may be strongly linked to these genes. Thus, WGCNA was employed to identify genes in modules that exhibited coexpression through synergistic expression. A soft threshold power of six was chosen (Figure 1C), resulting in the acquisition of eight coexpression modules (Figure 1D). DEGs were upregulated or downregulated in different modules, although MEpink contained only upregulated genes (Figure 1E and Supplementary Figure 1). Furthermore, the central gene of each module was determined (Table 1).

**TABLE 1:** Hub genes of the modules.

Color	Hub genes	Module
Black	BOP1	M6
Blue	PCK2	M7
Brown	SNRPD2	M8
Green	HCLS1	M3
Pink	NOTCH3	M1
Red	SCAMP3	M5
Turquoise	BUB1B	M2
Yellow	ECM1	M4

### Immunophenotype of patients with hepatocellular carcinoma

**FIGURE 2:**
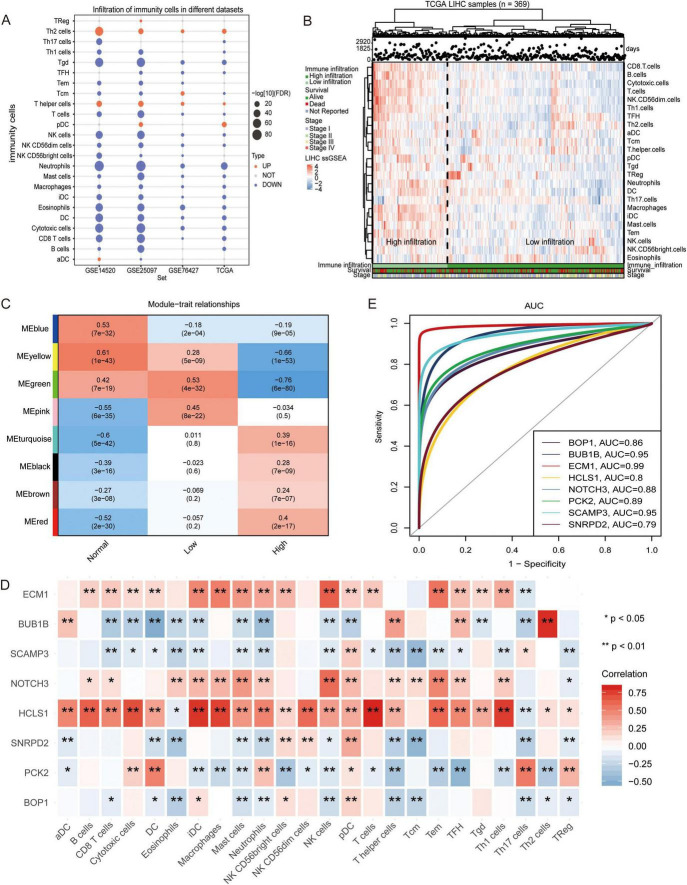
Infiltration of immune cells in hepatocellular carcinoma samples. **(A)** The change of immune cell composition between hepatocellular carcinoma (HCC) and control groups. Red indicates high infiltration, and blue indicates low infiltration. **(B)** The hepatocellular carcinoma samples were clustered into high immune cell infiltration and low immune cell infiltration groups using single-sample gene set enrichment analysis (ssGSEA) scores. The robustness of clustering was assessed using cophenetic correlation coefficient (*p* < 0.05). **(C)** The correlation between high and low immune infiltration groups was evaluated using Pearson correlation analysis. **(D)** Correlations between module hub genes and immune cells were assessed using Pearson correlation coefficient. **(E)** The Receiver Operating Characteristic curve for module hub genes was generated to evaluate their ability to distinguish between high and low immune infiltration groups. All the statistical significance was determined with *p* < 0.05. * indicates *p* < 0.05, ** indicates *p* < 0.01.

By detecting the difference in the proportions of the 24 immune cells between the HCC patients and the control individuals, we found that most of the immune cells in the HCC patients were decreased (Figure 2A), suggesting that the immune system may affect the occurrence of HCC. The infiltration fraction of immune cells in the TCGA cohort was detected using ssGSEA.

An unsupervised hierarchical clustering algorithm was used to cluster HCC samples into two groups (low infiltration and high infiltration) (Figure 2B). By calculating the correlation between the module and phenotype, MEyellow (module 4) was observed to have the highest positive correlation with the control, while MEgreen (module 3) had the highest positive correlation with low immune infiltration, and MEred (module 5) had the highest positive correlation with high immune infiltration (Figure 2C). Correlation analysis also revealed that HCLS1, the hub gene of module 3, exhibited a positive correlation with several immune cells, particularly T cells and DCs. SCAMP3, the hub gene of module 5, was negatively correlated with most immune cells, especially TCM cells (Figure 2D). The area under the ROC curve (AUC) values of the hub genes, as shown in Figure 2E, were greater than 0.79, with HCLS1 and SCAMP3 exhibiting values above 0.8.

### Biological functions and signaling pathways of the module genes

**FIGURE 3:**
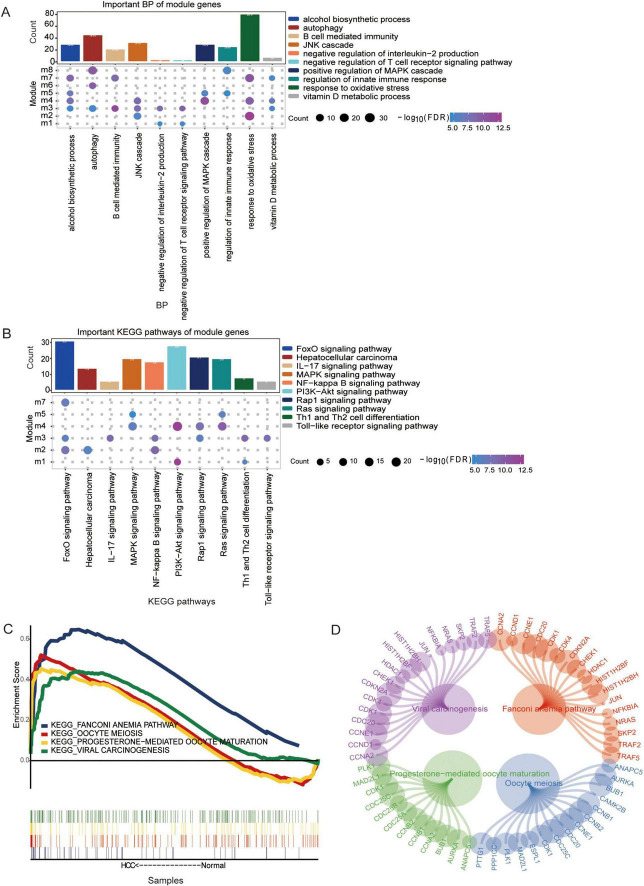
The biological functions and Kyoto Encyclopedia of Genes and Genomes (KEGG) pathways associated with the module genes are shown. **(A)** The biological process enriched for the module genes were determined using Gene Ontology (GO) enrichment analysis. **(B)** KEGG pathway enrichment analysis of the module genes. Pathways with *p* < 0.05 were considered significantly enriched. **(C)** The same KEGG pathway analysis of the module genes identified via GSEA and enrichment analysis. **(D)** The module genes involved in the same KEGG pathway were visualized using network analysis in Cytoscape.

Gene Ontology functional enrichment analysis revealed that the module genes were involved mainly in autophagy, the MAPK cascade, oxidative stress and other biological processes (BPs) (Figure 3A). Among them, module 3 genes were involved in the negative regulation of interleukin-2 production and the negative regulation of the T-cell receptor signaling pathway. The genes in Module 5 were involved in position regulation of the MAPK cascade, regulation of innate immune responses, and so forth. According to the KEGG enrichment results, the module genes were related mainly to the MAPK signaling pathway, FoxO signaling pathway, hepatocellular carcinoma pathway and other pathways (Figure 3B). In addition, four KEGG pathways were identified in the GSEA results and were equivalent to the enrichment results (Figure 3C) and the module genes involved in these signaling pathways (Figure 3D).

### Methylation factors regulate the prognosis of hepatocellular carcinoma

**FIGURE 4:**
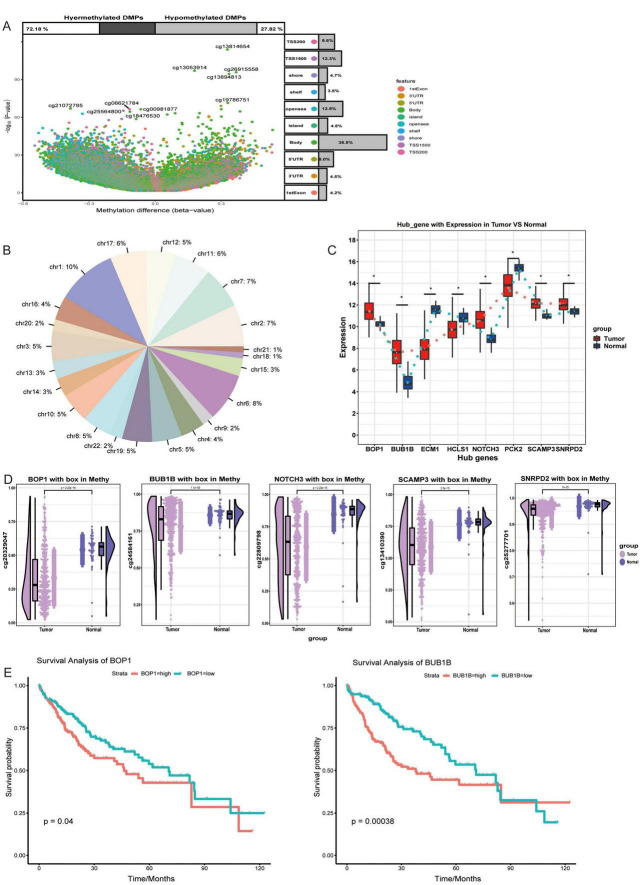
Changes in regulatory factors of hepatocellular carcinoma. **(A)** The methylation differences between hepatocellular carcinoma (HCC) patients and controls were assessed using Methylation Analysis (Illumina 450K BeadChip). Statistical significance was determined with *p* < 0.05. **(B)** The proportion of methylation sites across different genes was assessed and presented as pie plots. **(C)** The expression of hub genes between HCC and control groups. *p < 0.05. **(D)** The level of methylation of hub genes in the HCC and control groups. **(E)** K-M survival curve of genes that significantly influence overall survival, with *p* < 0.05 considered significant.

Next, the overall extent of methylation in HCC patients was investigated (Figure 4A), during which the proportion of methylation on different chromosomes was determined (Figure 4B). Among them, five hub genes (BOP1, BUB1B, NOTCH3, SCAMP3, and SNRPD2) were expressed at higher levels in HCC patients than in controls (Figure 4C). Interestingly, the hypomethylation of these genes occurs mainly in its promoter region. By comparing the methylation levels of HCC tissues with normal liver tissues, we found that the methylation of the promoter regions of these genes was significantly lower than that in normal controls, thus potentially leading to the upregulation of their transcriptional activity (Figure 4D). This finding suggested that these genes may be modified by methylation and may be methylation-driven genes. Survival analysis of patients stratified according to methylation status showed that BOP1 and BUB1B significantly affected OS in HCC patients (Figure 4E).

### Correlations of checkpoint molecules, BOP1 and BUB1B in HCC

**FIGURE 5:**
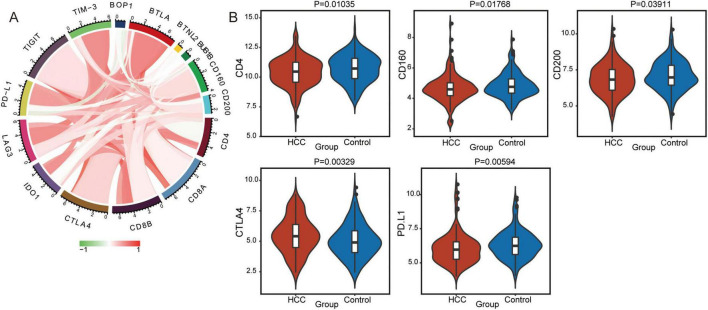
Immune checkpoint molecules between the hepatocellular carcinoma (HCC) and control group. **(A)** The correlations between BOP1 or BUB1B and immune checkpoint proteins were assessed using Pearson correlation analysis. Red indicates a positive correlation, and green indicates a negative correlation, with significance determined as *p* < 0.05. **(B)** The expression of immune checkpoint molecules between the HCC and control groups. *p* < 0.05 was considered significant.

Moreover, the correlations between immune checkpoint molecules and BOP1 or BUB1B were analyzed (Figure 5A). BOP1 was negatively correlated with PD-L1, CD4, CD160, and CD200, while BUB1B was positively correlated with TIGIT, CTLA4, and CD8A. Interestingly, immune checkpoint molecules were expressed at lower levels in HCC patients than in controls (Figure 5B). Therefore, BOP1 and BUB1B exhibit distinct correlations with immune checkpoint molecules, suggesting their potential roles in modulating immune responses in HCC, where immune checkpoint molecules are generally expressed at lower levels compared to controls.

## Discussion

In this study, we identified several DNA methylation-driven genes that significantly impact the prognosis of HCC patients, highlighting their potential as therapeutic targets. Our analysis integrated gene expression profiles and methylation data from multiple datasets to elucidate the complex interplay between genetic and epigenetic factors in HCC.

The identification of disease-related modules via WGCNA has become a powerful method for obtaining new insights into cancer biology (33–35). Each module may represent a different form of pathogenesis (36). The markers identified by WGCNA are closely related to HCC and have the potential to serve as diagnostic and prognostic markers for patients (37). Studies showed that key module genes and hub genes associated with poor prognosis of HCC were identified by WGCNA, including CCNB1, DLGAP5 (38) and ARPC4 (39). We identified eight coexpression modules through WGCNA, with specific focus on modules 3 and 5 due to their distinct immune infiltration profiles. Module 3, characterized by low immune infiltration, was significantly associated with the hub gene hematopoietic lineage cell-specific protein (HCLS1). HCLS1 showed a negative correlation with the infiltration of T cells and dendritic cells, suggesting an immunosuppressive role. A high expression level of HCLS1 in B-cell-derived malignant tumors was observed to be related to poor prognosis (40, 41). Moreover, the relationship between HCLS1 and HCC has rarely been reported, although it was negatively correlated with the OS of patients with colorectal cancer (42). HCLS1-associated protein X-1 (HAX-1) was observed to be significantly upregulated in human hepatoma tissues and demonstrated its ability to enhance cell proliferation (43).

Conversely, module 5 exhibited high immune infiltration and was associated with the hub gene secret carrier membrane protein 3 (SCAMP3). SCAMP3’s positive correlation with Tcm cells and its significant overexpression in HCC indicate its potential as an immune-related biomarker. Previous studies have identified high expression of SCAMP3 (44) as a poor prognostic marker in HCC, supporting our findings (45). The differential immune infiltration patterns observed in these modules underscore the heterogeneity of the HCC tumor microenvironment and highlight the necessity of personalized therapeutic strategies.

A small-scale clinical research institute conducted a systematic analysis and evaluated the efficacy of IL-2 in the treatment of HCC using the enrichment results of module 3, but the results were inconclusive (46). Further studies have indicated that IL-2 has the potential to enhance the antitumor activity of NK cells and inhibit the metastasis of HCC (47). T-cell receptor-mediated antigen-dependent tumor cytotoxicity directly induces cell death by binding to the Fas ligand on the membrane and suppresses tumor proliferation by secreting IFN-γ (48). TLRs potentially contribute to the inflammation-associated development and progression of HCC (49). Based on the enrichment results of module 5, the MAPK signaling pathway was found to be an important pathway that promotes the development of HCC (50). The occurrence and development of HCC are significantly influenced by innate immunity (51). In addition, the regulation of natural immune cells could enhance or serve as an alternative immunotherapy to overcome current limitations (52).

It is commonly believed that the activation of oncogenes or the inhibition of tumor suppressor genes could be attributed to abnormal hypermethylation and hypomethylation in cancer, as indicated by such epigenetic variations (53). Our study further identified five hub genes (BOP1, BUB1B, NOTCH3, SCAMP3, and SNRPD2) as methylation-driven. Among these, BOP1 and BUB1B were significantly associated with unfavorable overall survival, emphasizing their prognostic value. BOP1, known for its role in ribosome biogenesis, has been linked to advanced HCC and poor disease-free survival (54). Its upregulation, driven by hypomethylation, may contribute to tumor progression through enhanced cell proliferation. Similarly, BUB1B, a key player in the spindle checkpoint, was associated with high histological grade and vascular invasion in HCC, further corroborating its role in poor prognosis (55).

Methylation is usually associated with changes in gene expression, especially when methylation occurs in the promoter region (56). The low methylation mode may release the inhibitory effect of transcription factors on promoters, thereby promoting gene expression. We also found that the expression of these low methylation genes was significantly enhanced in HCC, indicating that the loss of methylation may be a key factor driving the overexpression of these genes during the disease process (57). The methylation status of these genes offers insights into potential therapeutic interventions. Targeting the epigenetic modifications that drive BOP1 and BUB1B expression could provide novel treatment avenues. Epigenetic therapies, such as DNA methyltransferase inhibitors, have shown promise in reversing abnormal methylation patterns and restoring normal gene function.

While the combination of existing methods might seem arbitrary, our integrated approach provides a comprehensive analysis of HCC from multiple dimensions—epigenetic, and immunological. To address concerns about reproducibility, we validated our findings across multiple datasets and ensured rigorous statistical controls.

## Limitations

This study has some limitations. Firstly, this study mainly relies on data from public databases for computational analysis, lacking direct experimental verification. Future research should further validate the biological functions of these hub genes and pathways through cell experiments and animal models. Secondly, in order to improve the reliability and universality of the results, future work will use independent clinical cohorts from different hospitals or databases for validation. In addition, although we integrated multiple datasets (TCGA, GSE14520, GSE25097, and GSE76427) and screened for common differentially expressed genes through intersection analysis, there may be some heterogeneity in sample sources, sample sizes, and experimental methods between different datasets. Despite our efforts to standardize and preprocess these data, sample heterogeneity may still affect the robustness of the results. The clinical translation of biomarkers still faces challenges, particularly in terms of accuracy, sensitivity, and specificity. Future research requires validation through large-scale clinical samples, combined with clinical data, to evaluate the potential applications of these biomarkers in early diagnosis, prognosis assessment, and personalized treatment of HCC. Due to the fact that this study mainly relies on secondary data analysis of public databases, certain potential confounding factors may affect the accuracy of the results. In future research, we will control for these potential confounding factors by designing more rigorous experimental validations.

## Conclusion

Our study highlights the significance of integrating genetic and epigenetic data to uncover novel therapeutic targets in HCC. HCLS1, SCAMP3, BOP1, and BUB1B are promising candidates for future research and clinical applications.

## Data Availability

The original contributions presented in this study are included in this article/Supplementary material, further inquiries can be directed to the corresponding authors.

## Footnotes

1
http://www.ncbi.nlm.nih.gov/geo/

2
https://portal.gdc.cancer.gov/repository

3
http://software.broadinstitute.org/gsea/index.jsp

4
http://www.bioinforcloud.org.cn
