# Supplementary figures and images for: DNA methylation-driven genes in hepatocellular carcinoma patients: insights into immune infiltration and prognostic implications
Source: Front Med (Lausanne). 2025 Apr 28;12:1520380. doi: 10.3389/fmed.2025.1520380 (PMC12066630; doi:10.3389/fmed.2025.1520380)

**Supplementary Material**

**Figure S1. Expression heatmap of module genes**


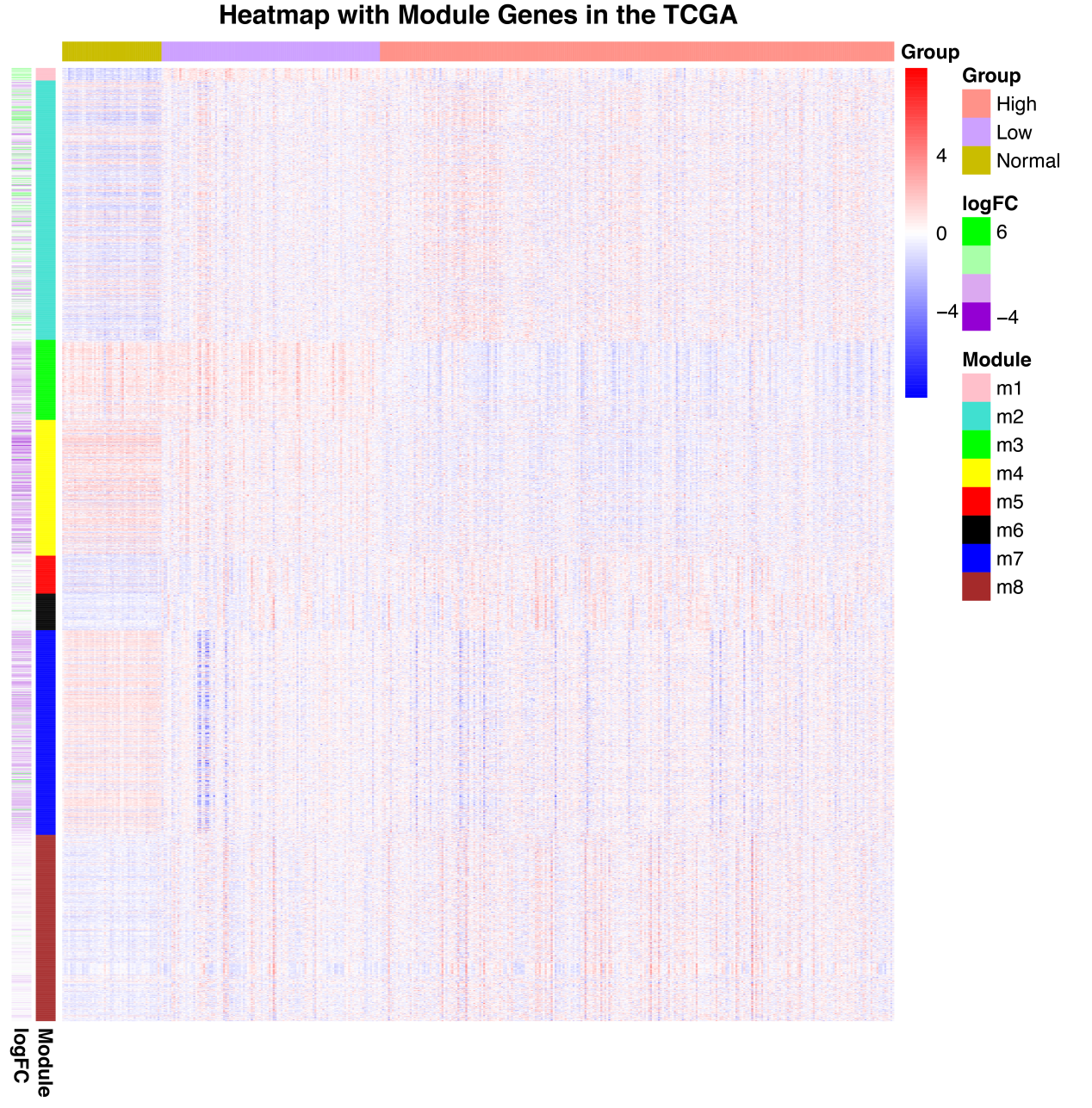

Supplement: Supplementary file 1 [file Supplementary_file_1.docx]
